# Exploring uncertainty measures in convolutional neural network for semantic segmentation of oral cancer images

**DOI:** 10.1117/1.JBO.27.11.115001

**Published:** 2022-11-03

**Authors:** Bofan Song, Shaobai Li, Sumsum Sunny, Keerthi Gurushanth, Pramila Mendonca, Nirza Mukhia, Sanjana Patrick, Tyler Peterson, Shubha Gurudath, Subhashini Raghavan, Imchen Tsusennaro, Shirley T. Leivon, Trupti Kolur, Vivek Shetty, Vidya Bushan, Rohan Ramesh, Vijay Pillai, Petra Wilder-Smith, Amritha Suresh, Moni Abraham Kuriakose, Praveen Birur, Rongguang Liang

**Affiliations:** a The University of Arizona, Wyant College of Optical Sciences, Tucson, Arizona, United States; b Mazumdar Shaw Medical Centre, Bangalore, Karnataka, India; c KLE Society Institute of Dental Sciences, Bangalore, Karnataka, India; d Mazumdar Shaw Medical Foundation, Bangalore, Karnataka, India; e Biocon Foundation, Bangalore, Karnataka, India; f Christian Institute of Health Sciences and Research, Dimapur, Nagaland, India; g University of California, Beckman Laser Institute & Medical Clinic, Irvine, California, United States; h Cochin Cancer Research Center, Kochi, Kerala, India

**Keywords:** uncertainty measures of deep learning, oral cancer, semantic segmentation, Monte Carlo dropout, Bayesian deep learning

## Abstract

**Significance:**

Oral cancer is one of the most prevalent cancers, especially in middle- and low-income countries such as India. Automatic segmentation of oral cancer images can improve the diagnostic workflow, which is a significant task in oral cancer image analysis. Despite the remarkable success of deep-learning networks in medical segmentation, they rarely provide uncertainty quantification for their output.

**Aim:**

We aim to estimate uncertainty in a deep-learning approach to semantic segmentation of oral cancer images and to improve the accuracy and reliability of predictions.

**Approach:**

This work introduced a UNet-based Bayesian deep-learning (BDL) model to segment potentially malignant and malignant lesion areas in the oral cavity. The model can quantify uncertainty in predictions. We also developed an efficient model that increased the inference speed, which is almost six times smaller and two times faster (inference speed) than the original UNet. The dataset in this study was collected using our customized screening platform and was annotated by oral oncology specialists.

**Results:**

The proposed approach achieved good segmentation performance as well as good uncertainty estimation performance. In the experiments, we observed an improvement in pixel accuracy and mean intersection over union by removing uncertain pixels. This result reflects that the model provided less accurate predictions in uncertain areas that may need more attention and further inspection. The experiments also showed that with some performance compromises, the efficient model reduced computation time and model size, which expands the potential for implementation on portable devices used in resource-limited settings.

**Conclusions:**

Our study demonstrates the UNet-based BDL model not only can perform potentially malignant and malignant oral lesion segmentation, but also can provide informative pixel-level uncertainty estimation. With this extra uncertainty information, the accuracy and reliability of the model’s prediction can be improved.

## 1 Introduction

Oral cancer is one of the leading causes of cancer-related deaths, especially in South Central Asia and Melanesia, accounting for 377,713 new cases and 177,757 new deaths in 2020, according to GLOBOCAN.^1^ It is highly prevalent in India, where the incidence rate in males was 13.9 per 100,000 and the mortality rate in females was 7.7 per 100,000 in 2018.^2^ In middle- and low-income countries such as India, the five-year survival rate is <50% due to late diagnosis, according to the paper published in 2014.^3^ Therefore, point-of-care screening platforms and algorithms are in great need.

Two important deep-learning applications in clinical research are accurate and automated medical image classification and segmentation. Deep learning has achieved state-of-the-art performance in medical image analysis, including classification and segmentation for cancer diagnosis. Many deep-learning methods have been used to perform the diagnosis of different cancers, including skin cancer,^4^ breast cancer,^5^ and oral cancer.^6^ Despite the state-of-the-art performance of deep learning in medical segmentation, it rarely provides an uncertainty estimation when making predictions. Deep-learning models are often considered black boxes due to a lack of theoretical understanding of their underlying mechanisms. To improve the reliability of deep-learning methods and use them for clinical applications, uncertainty estimation related to the model’s prediction is a key factor to consider. The Bayesian deep learning (BDL) model^7^ provides a framework to accomplish this task by modeling the posterior distribution. Bayesian networks learn a distribution over their weights instead of deterministic ones.

Some researchers have used BDL for different medical applications to quantify uncertainty. Liu et al.^8^, introduced deep spectral learning for optical imaging oximetry with uncertainty quantification. Chai et al.^9^ proposed a Bayesian deep multisource learning model that incorporates model uncertainty into glaucoma diagnosis. Rączkowski et al.^10^ introduced a Bayesian convolutional neural network (CNN) for classifying histopathological colorectal images with uncertainty measurements. Liu et al.^11^ designed a spatial attentive BDL network for automatic segmentation of the peripheral and transition zones of the prostate with uncertainty estimation. Some pioneering works have also applied BDL to build more reliable deep-learning methods for diagnosis of diseases including, but not limited to, skin cancer,^12^ oral cancer,^13^ and prostate cancer.^14^

In general, BDL research for medical applications is an active topic aimed at improving the robustness and reliability of CNNs. Therefore, we introduce an uncertainty estimation method for oral cancer image segmentation based on a Bayesian UNet architecture in this study. The dataset used in this study contains 492 white-light images that were captured using our customized oral cancer screening platform^15^^,^^16^ and annotated by oral oncology specialists. There are several different types of oral potentially malignant disorders that have unique clinical features that can be observed under white-light illumination.^17^ Nonhomogeneous leukoplakia is one such disorder that commonly includes symptoms of white and/or red patches; small polypoid outgrowths; rounded, red, or white excrescences; and a wrinkled or corrugated surface appearance. The lesions of erythroplakia are usually irregular in outline and have a bright red velvety surface. Because these unique clinical features are the basis for diagnosis, machine learning algorithms need to find them in images to perform correct automatic diagnosis.^17^ We trained the model and evaluated the segmentation and uncertainty estimation performance using multiple metrics. We also compared models with MC dropout layers applied to only the contracting path or expansive path, or both, and built an efficient model by replacing the convolutional layers with separable convolutional layers. To the best of our knowledge, this is the first study leveraging BDL to enhance the reliability and understandability of results from deep learning-based oral cancer image segmentation.

## 2 Material and Methods

### 2.1 UNet Architecture

Multiple deep neural network architectures have been proposed for medical image segmentation. In this study, we used UNet as the base network. UNet^18^ consists of a contracting path and an expansive path. The contracting path contains multiple contracting blocks, wherein each block has two 3×3 convolutions, followed by a rectified linear unit (ReLU) and a 2×2 max pooling operation with a stride of 2 for downsampling. The number of feature channels doubles at each downsampling step. The expansive path contains multiple expansive blocks, and each block has a 2×2 up-convolution that halves the number of feature channels, a concatenation with the correspondingly cropped feature map from the contracting path, and two 3×3 convolutions followed by a ReLU.

Because the uncertainty estimation of Bayesian architecture needs multiple time inferences (discussed in Sec. 2.2), it may need more computing time and resources. In this study, we replaced the conventional convolutional layers in the UNet with more efficient depthwise separable two-dimensional convolution layers.^19^ A depthwise separable convolution layer is small, has low latency, and has low power consumption, all characteristics that allow it to meet the needs of real-time high accuracy analysis for on-device embedded applications. Depthwise separable convolution layers include a depthwise convolution and a pointwise convolution; the depthwise convolution layer filters each of the input channels, and the pointwise convolution layer combines the results through the depthwise convolution layer. This conversion reduces both the computational cost and model size. The computational cost of standard convolution is $D_{f}\times D_{f}\times M\times N\times D_{k}\times D_{k}$, where $D_{f}$ is the spatial width and height of the input feature map, M is the number of input channels, $D_{k}$ is the spatial dimension of the kernel, and N is the number of output channels. However, the computational cost of the depthwise separable convolution is $D_{f}\times D_{f}\times M\times D_{k}\times D_{k}+D_{f}\times D_{f}\times M\times N$. By converting standard convolution to the depthwise separable convolution, the computational cost is reduced by a factor of $(D_{f}\times D_{f}\times M\times D_{k}\times D_{k}+D_{f}\times D_{f}\times M\times N)=1/N+1/D_{k}^{2}$.

### 2.2 Bayesian Deep Learning

Despite their success in different medical tasks, one of the limitations of CNNs for medical applications is their inability to provide prediction uncertainties. The softmax output (predictive probabilities) obtained at the end of a CNN is often erroneously interpreted as model confidence. This is an unwise solution, however, as a model can be uncertain in its prediction even with a high softmax output. Uncertainty quantification is a key factor for the clinical application of deep learning methods because it can increase the reliability of results provided by these methods. BDL models provide a framework for estimating uncertainty by modeling the posterior distribution.^20^^–^^22^

**Fig. 1:**
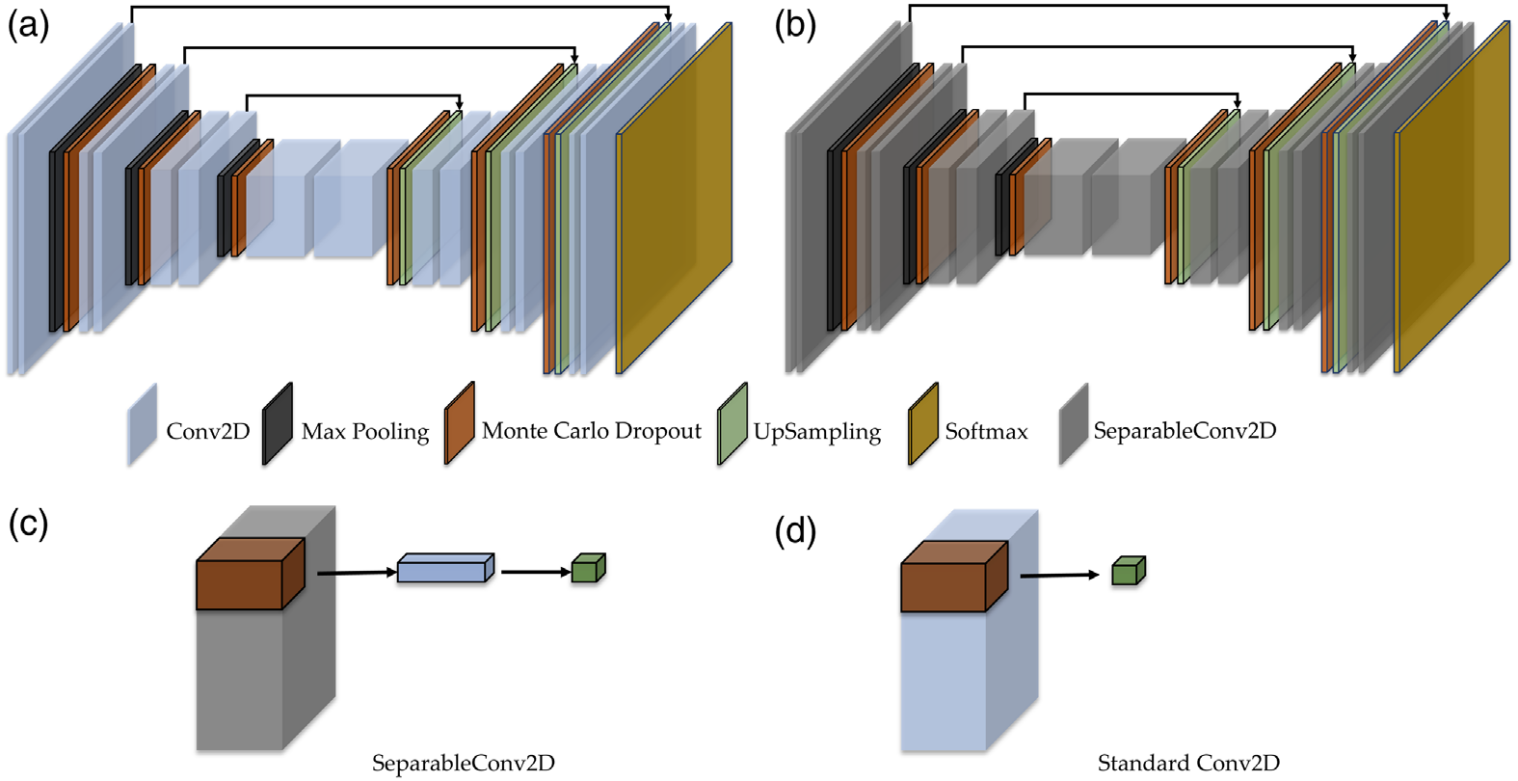
The proposed BDL model for oral cancer image segmentation based on UNet with (a) conventional convolutional layers; (b) efficient depthwise separable convolution layers; (c) depthwise separable convolution filter; and (d) standard convolution filter.

Bayesian networks are probabilistic models, not deterministic ones, that learn a distribution over their weights. Given training data X and Y, they aim to learn the posterior distribution of the neural network’s weights W. The posterior distribution is often approximated using variational inference methods, such as Dropout variational inference. Monte Carlo (MC) dropout^23^ can be considered using the Bernoulli distribution to approximate distributions over the network’s weights. The prediction distribution of a Bayesian deep network for a new input x* is modeled as

$$p(y^{*}|x^{*},X,Y)=\int p(y^{*}|x^{*},W)p(W|X,Y)dW,$$

where $p(y^{*}|x^{*},W)$ is the Softmax function and p(W|X,Y) is the posterior over the weights. The prediction is approximated by sampling the model multiple (σ) times. The uncertainty is obtained by calculating the variance:

$$p(y^{*}|x^{*},X,Y)\approx \frac{1}{\sigma }\overset{\sigma }{\underset{i=1}{\sum }}\mathrm{Softmax}(fW_{i}^{*}(x^{*})),$$

$$v=\;\frac{1}{\sigma }\overset{\sigma }{\underset{i=1}{\sum }}{\left(p(y|x^{*},W_{i})-p(y|x^{*},X,Y)\right)}^{2},$$

where $p(y|x^{*},W_{i})$ represents σ times softmax output with different weights $W_{i}$ of input $x^{*}$ and $p(y|x^{*},X,Y)$ is the predictive posterior mean of input $x^{*}$. This study applied MC dropout layers (with 0.5 rate) in each contracting block, in each expansive block, or in both paths simultaneously. The MC Dropout layer was placed following the max pooling layer in the contracting block, whereas in the expansive block, the MC Dropout layer was placed following the up-convolutional layer. The proposed UNet-based BDL model is shown in Fig. 1(a), which is the original model with conventional convolutional layers, and Fig. 1(b) shows the efficient model with depthwise separable convolution layers.

### 2.3 Dataset

**Fig. 2:**
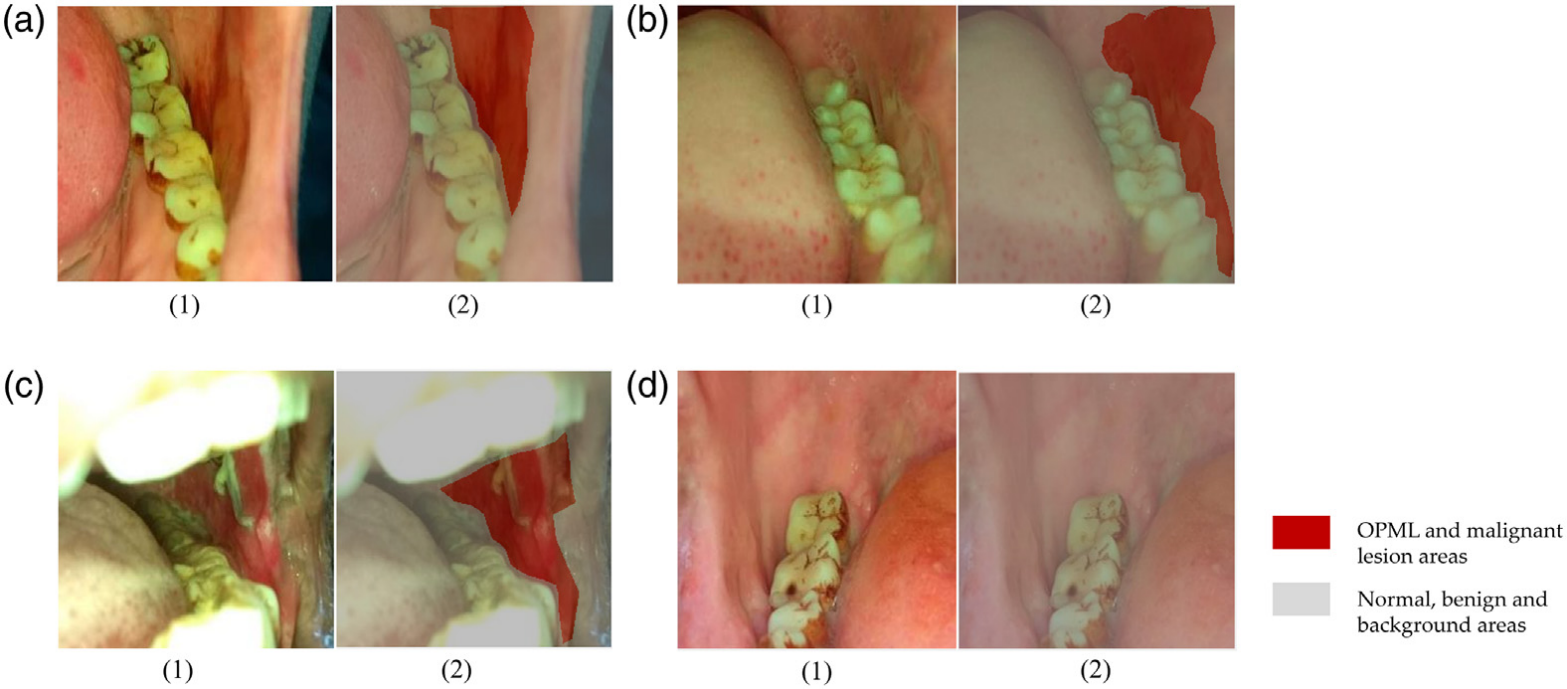
Examples of the dataset used for this study. (a1)–(d1) White-light oral cavity images captured using our customized oral cancer screening platform. (a2)–(d2) Corresponding pixel-level annotations labeled by oral oncology specialists. (a)–(c) Three positive samples and (d) one negative sample. The OPML and malignant lesion areas are shown in red, and other areas are shown in gray.

The dataset used in this study contains 492 white-light images that were captured using our customized oral cancer screening platform^15^^,^^16^ from patients attending the outpatient clinics of the Department of Oral Medicine and Radiology at the KLE Society Institute of Dental Sciences, the Head and Neck Oncology Department of Mazumdar Shaw Medical Center, and the Christian Institute of Health Sciences and Research, India. Institutional ethics committee approval was obtained from all participating hospitals and written informed consents were collected from all subjects enrolled. These images were annotated by oral oncology specialists from Mazumdar Shaw Medical Center, KLE Society Institute of Dental Sciences, and Christian Institute of Health Sciences and Research using MATLAB Image Labeler.^24^ The oral potentially malignant lesion (OPML) and malignant lesion areas in these images were labeled. The dataset used in this study contains 396 positive samples that have OPML and malignant lesions and 96 negative samples (examples shown in Fig. 2). We performed 10-fold cross-validation in this study.

### 2.4 Evaluation Metrics

Intersection over union (IoU) and pixel accuracy were used as evaluation metrics for segmentation performance. For each class, IoU is the ratio of correctly classified pixels to the total number of ground truth and predicted pixels in that class. Mean IoU is the average IoU score of all classes, and weighted IoU is weighted by the number of pixels in each class. Pixel accuracy is the ratio of correctly classified pixels to the total number of pixels in that class according to the ground truth.

Whereas segmentation performance evaluation is straightforward using IoU and pixel accuracy, uncertainty performance evaluation for segmentation is more challenging because it is hard to define a good uncertainty estimate. Mukhoti and Gal^25^ proposed two intuitive desiderata to define a good uncertainty estimation: (1) if a model is confident about its prediction, it should be accurate on the same and (2) if a model is not confident about its prediction, it may or may not be accurate. Based on these two desiderata, they put forward two conditional probabilities as uncertainty evaluation metrics, p (accurate | certain) and p (uncertain | inaccurate), and the combination of them, patch accuracy versus patch uncertainty (PAvPU). P (accurate | certain) is the probability that the model is accurate on its output, given that it is confident on the same. P (uncertain | inaccurate) is the probability that the model is uncertain about its output, given that it has made a mistake in its prediction. PAvPU combines both the (accurate, certain) and (inaccurate, uncertain) patches into a single metric.

In this study, we used their proposed metrics to evaluate the uncertainty estimation performance of our oral cancer segmentation models. To calculate these metrics, we traversed the predicted labels, ground truth labels, and uncertainty maps using windows of 2×2 in size. A binarized accuracy map was obtained by computing the accuracy of each patch from the predicted and ground truth labels. If the patch accuracy is higher than 0.5, it is flagged as accurate; otherwise, it is flagged as inaccurate. Similarly, the average patch uncertainties were computed from the uncertainty map. If the PAvPU value is above a certain threshold, it is flagged as uncertain; otherwise, it is flagged as certain. The patch accuracy threshold and uncertainty threshold are both tunable parameters; we fixed the patch accuracy value as 0.5 and allowed the uncertainty threshold to vary. In this study, the pixel uncertainty values were first normalized to [0.0 1.0] for subsequent calculations. Next, we counted the number of patches, which are accurate and certain ($n_{ac}$), accurate and uncertain ($n_{au}$), inaccurate and certain ($n_{ic}$), and inaccurate and uncertain ($n_{iu}$). The evaluation metrics are then calculated as

$$p(\mathrm{accurate}|\mathrm{certain})=\frac{n_{ac}}{n_{ac}+n_{ic}},$$

$$p(\mathrm{uncertain}|\mathrm{inaccurate})=\frac{n_{iu}}{n_{ic}+n_{iu}},$$

$$\mathrm{PAvPU}=\frac{n_{ac}+n_{iu}}{n_{ac}+n_{au}+n_{ic}+n_{iu}\;}.$$

## 3 Experiments and Results

The training data was augmented multiple (n=6) times with horizontal/vertical flipping, rotation, zoom, brightness adjustment, and gamma correction. The Adam optimizer was used with a batch size of 16 in each experiment. All models were trained for 300 epochs, and the best model was saved after every epoch if there was a decrease in validation loss. Code implementation was made with Keras and Tensorflow backend (using the Python programming language), and the training was done on the high-performance computing platform of the University of Arizona.^26^ The trained models were inferenced on a desktop computer with an Intel Xeon Silver 4114 CPU, an Nvidia 1080Ti GPU, and 32 GB of RAM. For uncertainty estimation, the models were sampled multiple (σ=100) times for each test image.

First, we trained a network with MC dropout layers applied to each contracting and expansive block using conventional convolutional layers. We evaluated the Bayesian deep neural network’s segmentation performance with 10-fold cross-validation and compared it with an original UNet model without any MC dropout layers (see Table 1). The model achieved 0.714 mean IoU, 0.796 weighted IoU, and 0.881 pixel accuracy, and it performed better than the original UNet model on the oral dataset. These results show that this model was able to segment the oral potential malignant lesion and malignant lesion areas from healthy tissue and background.

**Table 1:** Segmentation performance comparison of the Bayesian deep-learning network with original UNet (mean and standard deviations of the cross-validation).

	Pixel accuracy	Mean IoU	Weighted IoU	Dice similarity coefficient
MC dropout network	0.881 (0.012)	0.714 (0.010)	0.796 (0.017)	0.733 (0.009)
Original UNet	0.855 (0.008)	0.698 (0.013)	0.736 (0.015)	0.706 (0.007)

**Fig. 3:**
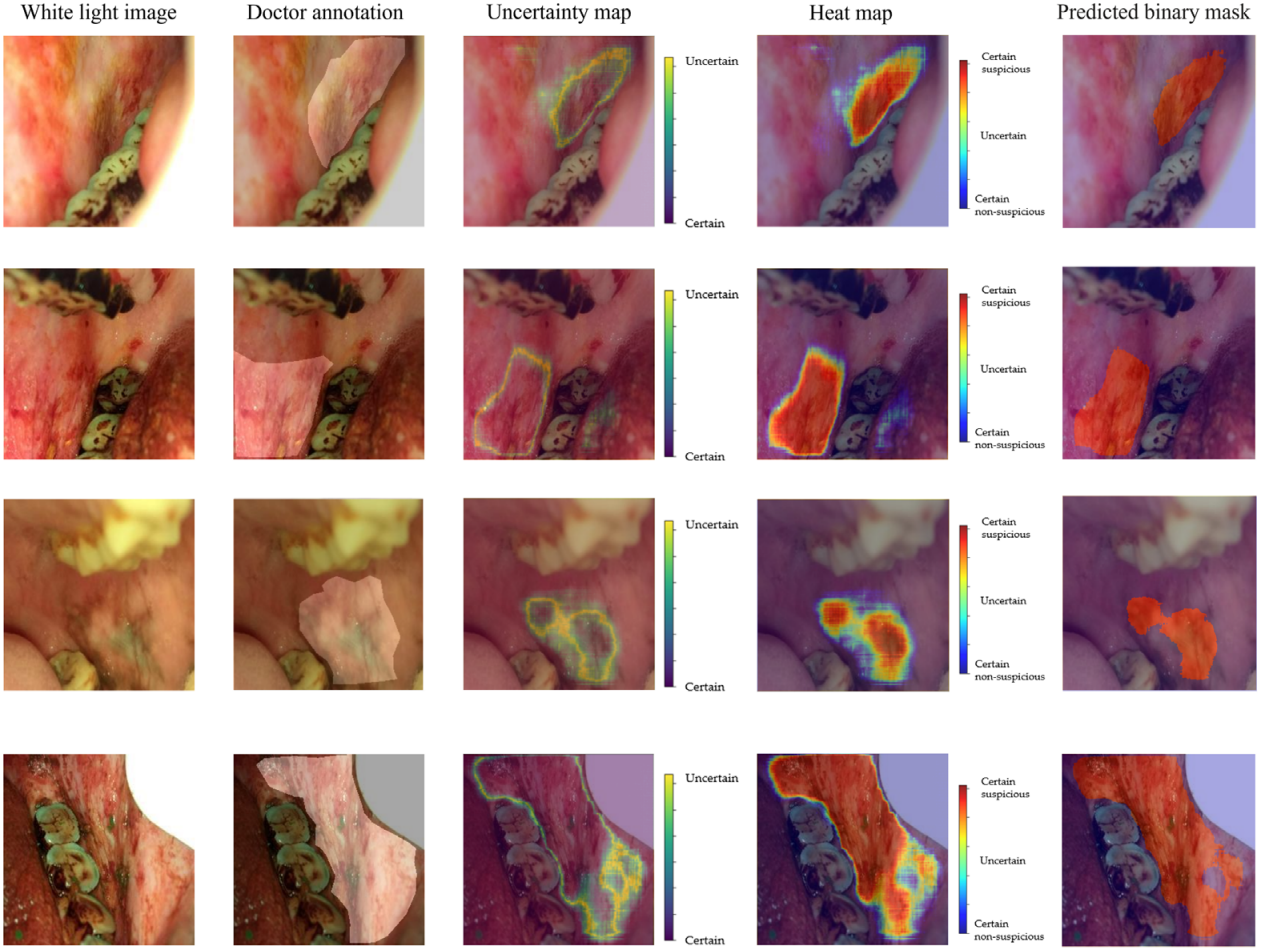
Example results of uncertainty estimation and lesion segmentation using the proposed Bayesian deep learning model for oral cancer image segmentation. The first column shows the original white-light images, the second column shows the annotation by specialists, the third column shows the uncertainty estimation generated by the model, the fourth column shows the uncertainty estimation and lesion segmentation together in the form of a heatmap, and the fifth column shows the predicted binary label mask.

Figure 3 shows examples of model predictions that include uncertainty estimations. Figures 3(a), 3(e), and 3(i) are three white-light oral cavity images. Figures 3(b), 3(f), and 3(j) show the doctor’s annotations. Figures 3(c), 3(g), and 3(k) present examples of uncertainty estimation of these cases. These uncertainty maps are obtained by sampling 100 predictions from the model and estimating the standard deviation for each pixel. Pixels displayed in bright green are associated with high uncertainty, and pixels displayed in dark blue are associated with high certainty. Figures 3(d), 3(h), and 3(l) show the results of uncertainty estimation as well as lesion segmentation. These heatmaps are obtained by combining the information of segmentation and uncertainty estimation. Pixels displayed in red are associated with high certainty of suspicious lesion areas, pixels displayed in dark blue are associated with high certainty of non-suspicious areas, and pixels displayed in green are associated with high uncertainty.

The cases shown in the first two rows of Fig. 3 indicate that the model has high confidence for most pixels in its prediction, except for pixels near lesion borders. This is reasonable as it is difficult to assess the lesion edges accurately even for experienced specialists. In addition to the edges, the model also shows uncertainty with (1) some suspicious areas that are not obvious and (2) some non-suspicious areas with feature changes. Indeed, these are the extra pieces of information that we expect the uncertainty estimation to divulge to help find challenging prediction areas. For example, although the model fails to segment parts of the suspicious areas in the last case (the last row of Fig. 3), the model shows high uncertainty on suspicious areas that are not obvious and low uncertainty on more obviously suspicious areas. These examples indicate the BNN model can produce pixel-level uncertainty estimation.

**Fig. 4:**
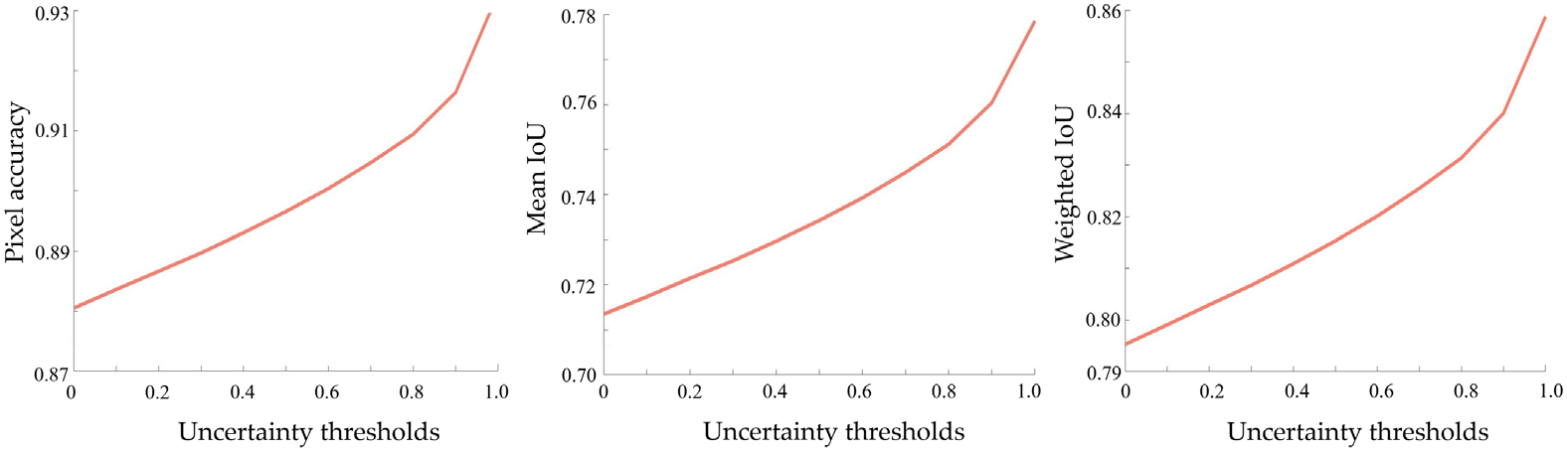
The change of pixel accuracy, mean IoU, and weighted IoU when removing pixels with uncertainty values higher than a specific level.

By removing some of the uncertain pixels and leaving these confusing areas for further inspection, the model can produce more accurate and reliable segmentation results on the remaining areas. We measured the change in pixel accuracy, mean IoU, and weighted IoU when removing pixels with uncertainty values higher than a specific level. By adjusting the level of uncertainty thresholding, we plotted the change of these three evaluation metrics in Fig. 4. We can see a continuous increase in all three evaluation metrics in response to a change in uncertainty thresholding.

**Fig. 5:**
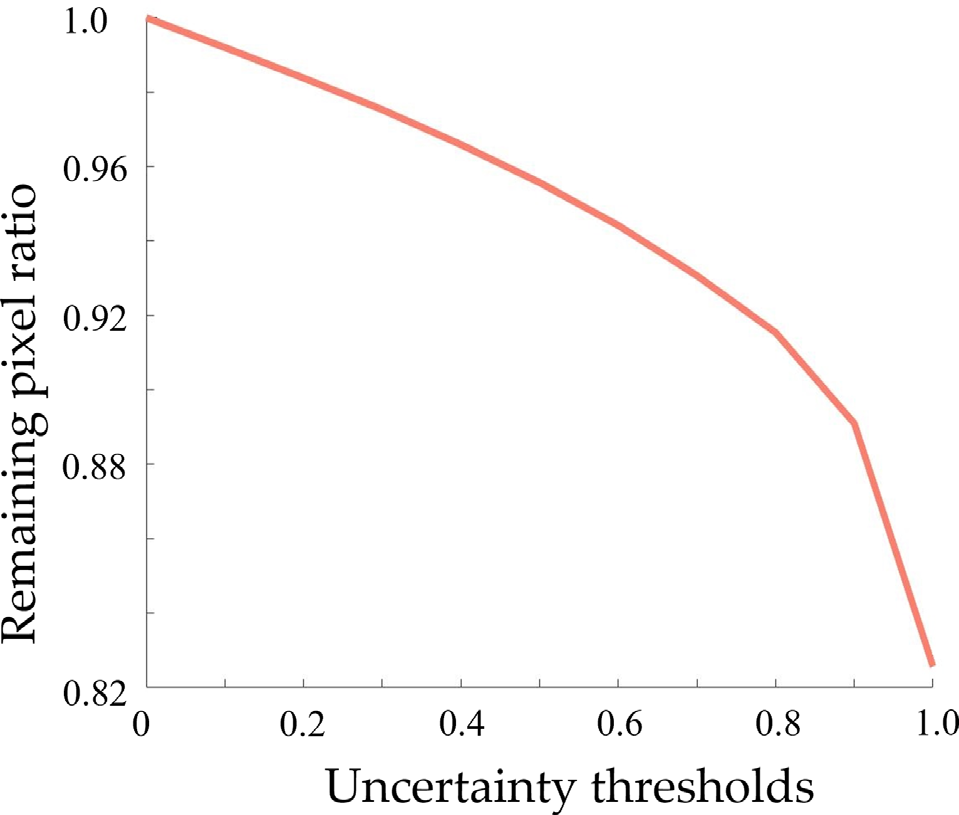
The remaining pixel ratios (1—removed pixel ratio) corresponding to different uncertainty thresholds.

To check if this process removed too many uncertain pixels, we monitored the remaining pixel ratios change (1—removed pixel ratio) corresponding to different uncertainty thresholds (see Fig. 5). If we want ∼90% of the pixels to remain, the model can achieve a pixel accuracy of 0.911, a mean IoU of 0.751, and a weighted IoU of 0.841. This is higher than 0.881/0.714/0.796, the result without removing uncertain pixels (see Table 2). This experiment was not trying to prove that this method could improve the accuracy by removing some uncertain pixels. The result only demonstrated that the model provides less accurate predictions in uncertain areas and may need more attention and further inspection. We expect the proposed method could provide extra pieces of uncertainty information in addition to the binary segmentation result to help find challenging prediction areas that need further inspection. The removed 10% pixels (uncertainty pixels as described above) were located mainly at the lesion boundary, and some suspicious areas that are not obvious as well as some non-suspicious areas with feature changes. This coincides with our expectations, as it is difficult to assess the lesion edges accurately even for experienced specialists.

**Table 2:** Segmentation performance comparison of the Bayesian deep learning network with and without removing uncertain pixels (mean and standard deviations of the cross-validation).

	Pixel accuracy	Mean IoU	Weighted IoU
MC dropout network	0.881 (0.012)	0.714 (0.010)	0.796 (0.017)
After removing part of uncertain pixels (90% pixels remain)	0.911 (0.016)	0.751 (0.019)	0.841 (0.012)

**Fig. 6:**
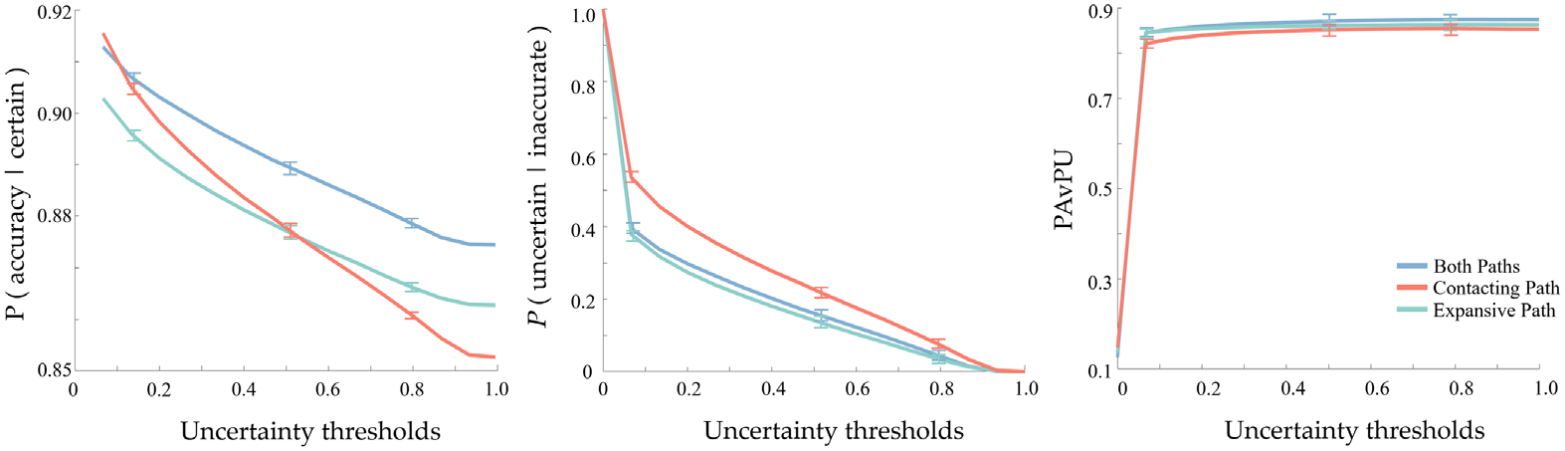
Uncertainty estimation performance comparison of three models by adding MC dropout layers on contracting or expansive blocks or both, using p (accurate | certain), p (uncertain | inaccurate), and patch accuracy versus PAvPU.

Because we were also curious about the influence of MC dropout layers when applied to the contracting or expansive paths of UNet, we trained two more models that either (1) only add MC dropout layers in the contracting blocks or (2) only add MC dropout layers in the expansive blocks. These two models were trained using the same parameter settings and with 10-fold cross-validation. The segmentation performance comparison is shown in Table 3. The segmentation performance of the first model is the best, which may be due to more dropout layers resulting in less overfitting. To evaluate the uncertainty estimation performance, we calculated and compared the p (accurate | certain), p (uncertain | inaccurate), and PAvPU described in Sec. 2.4. The comparison is shown in Fig. 6. The model with MC Dropout layers applied to both contracting and expansive paths works better than the other two models on p (accurate | certain) and PAvPU, whereas the model with MC dropout layers applied only to contracting path works better than the other two models on p (uncertain | inaccurate). For Fig. 6, the values of these metrics depended on three parameters (described in Sec. 2.4): the patch dimensions, the accuracy threshold, and the uncertainty threshold. We fixed the patch dimensions as 2×2 and the accuracy threshold as 0.5. We then observed how these metrics varied with a change of uncertainty threshold. A model with a higher value of these metrics is a better performer.

**Table 3:** Segmentation performance comparison of three models by adding MC dropout layers on contracting blocks, expansive blocks, or both (mean and standard deviations of the cross-validation).

	Pixel accuracy	Mean IoU	Weighted IoU	Dice similarity coefficient
MC dropout added on all contracting and expansive blocks	0.881 (0.012)	0.714 (0.010)	0.796 (0.017)	0.733 (0.009)
MC dropout added on contracting blocks only	0.862 (0.008)	0.693 (0.013)	0.775 (0.014)	0.702 (0.003)
MC dropout added on expansive blocks only	0.851 (0.006)	0.702 (0.012)	0.750 (0.009)	0.725 (0.005)

**Fig. 7:**
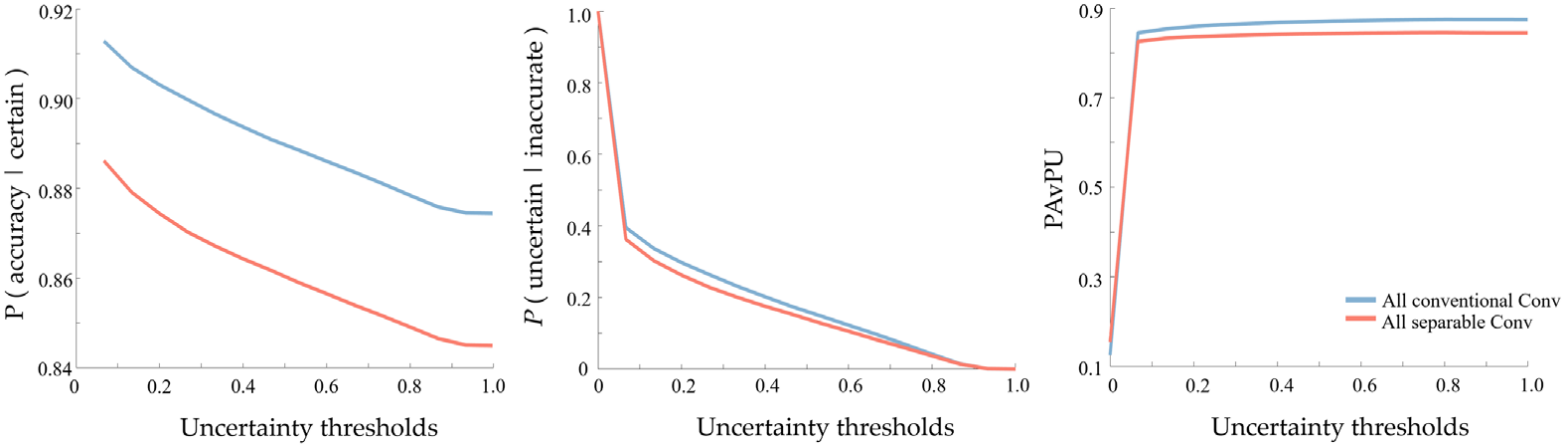
Uncertainty estimation performance comparison of the efficient and original models, using p (accurate | certain), p (uncertain | inaccurate), and patch accuracy versus PAvPU.

Although the computing time of one single inference is insignificant, the models need to be sampled multiple times to estimate the uncertainty, so efficiency is a concern, especially for the outdated computers and portable devices commonly used in resource-limited settings. Therefore, we built a more compact and efficient model by replacing the convolutional layers of the original model with depthwise separable convolutional layers (described in Sec. 2.1). The new model was trained using the same settings and with 10-fold cross-validation. The efficient model is almost six times smaller than the original one (3.42 MB versus 20 MB). The computing speed when sampling 100 times is also faster using the desktop computer mentioned before (42 s versus 1 min 24 s). These improvements make our new efficient model more suitable for future implementation on portable devices. We were worried about whether the improvement of efficiency might affect segmentation and uncertainty estimation performance, so we also calculated the mean IoU, weighted IoU, and global pixel accuracy, as well as p (accurate | certain), p (uncertain | inaccurate), and PAvPU and compared with the original model. The results are shown in Table. 4 and Fig. 7. This experiment shows that the efficient model reduced the computation time and size, albeit with some performance compromises. Although the performance compromises of the efficient model are not huge, the difference in accuracy is still important and not negligible for rigorous medical applications such as a treatment protocol design. Therefore, the efficient model will be considered for detection tasks in resource-limited settings. We are interested in implementing the efficient model on portable devices and comparing the performance with a model directly distilled from the UNet to be smaller.

**Table 4:** Segmentation performance comparison of the efficient and original models (mean and standard deviations of the cross-validation).

	Pixel accuracy	Mean IoU	Weighted IoU	Dice similarity coefficient
All convolutional layers are conventional convolutional layers	0.881(0.012)	0.714(0.010)	0.796 (0.017)	0.733 (0.009)
All convolutional layers are separable convolutional layers	0.850 (0.010)	0.638 (0.008)	0.771 (0.013)	0.695 (0.007)

## 4 Conclusion

In this paper, we have proposed a Bayesian UNet architecture for uncertainty estimation of oral cancer image segmentation and have shown that the model achieves good segmentation accuracy with 10-fold cross-validation. By sampling the model multiple times, uncertainty maps of oral cancer images can be obtained. The uncertainty maps can provide more pixel-level information than segmentation predictions alone. From the results, we observe that the model is uncertain with (1) lesion borders, (2) some suspicious areas that are not obvious, and (3) some non-suspicious areas with feature changes when making the prediction. With this extra uncertainty information, the accuracy and reliability of the model’s prediction can be improved. In the experiments, we observed an improvement in pixel accuracy and mean IoU by removing uncertain pixels. This result reflects that the model provides less accurate predictions in uncertain areas that may need more attention and further inspection. To evaluate the segmentation uncertainty estimation of our models, we also used the metrics introduced by Mukhoti and Gal.^25^ We experimentally compared three models with MC dropout layers applied to only the contracting path, to only the expansive path, and to both paths simultaneously. We also built and tested an efficient model by replacing the conventional convolutional layers with depthwise separable convolutional layers. The efficient model is almost six times smaller and two times faster than the original UNet, with small performance compromises, expanding its potential for future implementation on portable devices. In general, our proposed method can effectively segment the OPML and malignant lesion areas from healthy tissue and background, as well as estimate uncertainty when making the prediction.
